# The National Hospital

**Published:** 1900-07-14

**Authors:** 


### The Hospital

A Journal of

The Medical Sciences and Hospital Administration.

Vol. XXVIII.—No. 720.] Edited by Sir Henry Burdett, K.C.B., and Solomon C. Smith, M.D., M.R.C.P. [July 14, 1900.

### THE NATIONAL HOSPITAL.

The circular addressed to the governors of the National Hospital for the Paralysed and Epileptic, to which we made a brief reference recently, is really one which opens a very wide and very important question of hospital management. The authors of the circular, or, in other words, the whole of the members of the medical and surgical staff of the institution, say, in effect, that the government of the hospital, or of any hospital, can only be effective when it is controlled by medical knowledge; and that persons destitute of this knowledge, however zealous or well-meaning, cannot be preserved from liability to fall into errors which may be serious in their consequences. The contention is an old one, but it derives great additional force from the extent to which, in recent times, the conception of medical or surgical treatment has gone beyond the mere performance of operations or the administration of remedies, and has been extended to cover almost the whole of life.

Such details as the temperature of the wards, the hours of rising and of lying down, the times for meals and the length of the intervals separating them, the opening or shutting of windows, the admission or exclusion of visitors, the regulation of both bed and body clothing, and a hundred other things of like kind, which, only a few years ago, would have been regarded as in the main "domestic" matters, which might safely be left to a matron or housekeeper, have now assumed positions of considerably enhanced importance, and may, it is pointed out, exert a very material influence upon the welfare of the patients. For example, one of the questions mentioned in the circular has reference to an alleged insufficiency of draw sheets, which, especially in the case of the paralysed, would manifestly entail an increased liability to the formation of bedsores. It is further alleged that these sheets, when wetted, were frequently not washed, but merely dried to prepare them for renewed use—an arrangement which might not only promote the formation of bed-sores, but also open the way for the septic infection of wounds, whether these were surgical or accidental. We know quite well that, in private life, a physician or surgeon who was unable absolutely to control the management of the sick-room would best consult his reputation by retiring from the case; and it seems manifest that a rule which would be of universal application in a family house should be observed with equal stringency in a hospital. If, after the doctors were gone, the house mistress were to give a series of directions either antagonistic to theirs, or framed in total disregard or ignorance of them, it is manifest that the position would soon become so much strained as to be unbearable.

It was formerly a popular allegation, carrying much weight at the meetings, especially of provincial hospitals, that doctors were not "business-like," and that, out of compassion for the presumed incompetence hence arising, it would be charitable to relieve them from administrative responsibilities which they would not be prepared adequately to discharge. Very often, no doubt, the doctors, while laughing in their sleeves at the reason assigned, were not sorry to obtain immunity from troublesome duties; but the case altogether changes when the discharge of these duties becomes a matter influencing the results of treatment. In such circumstances it is necessary to speak with no uncertain voice; and to demand, not merely as a matter of policy, but as a matter of right, that, in accordance with the old proverb, the chamber of the sick man should be the kingdom of the physician.

The medical staff are the persons primarily responsible for the success of the work which it is their business to carry to a completion; and they cannot, consistently with their duties and obligations to the public, permit the success of this work to be jeopardised by ill-judged parcimony or by absurd "domestic regulations." Some of these, no doubt, would be designed as measures of economy, in absolute ignorance that their adoption might mean the loss of human lives. Besides these manifest considerations, which appear to us fully to justify the principle of that demand for adequate representation on the Board of Management on which the matters in dispute seem now to turn, there is the further valid consideration that the hospital has lately been thrown open by payment, without, in the opinion of the staff, any sufficient security against the systematic abuse of the privilege. In the present state of professional feeling as to the extent to which hospitals deprive general practitioners of due remuneration for their services, it is plain that no staff of men engaged in consulting practice can fairly be asked to set this feeling at defiance, and to be held responsible for what, in many cases, is alleged to amount to a grave evil. The staff, therefore, are contending for representation for two chief purposes, first, that their knowledge of the requirements of the sick may be utilised for the purpose of securing the efficient treatment of the patients, and next, that proper inquiries into alleged poverty may be instituted, before the allegation is accepted as a passport for treatment at a merely nominal charge.

